# The Incidental Use of High-Dose Vitamin D3 in Pancreatic Cancer

**DOI:** 10.1089/crpc.2016.0003

**Published:** 2016-05-01

**Authors:** Timothy L. Cannon, Joel Ford, Danubia Hester, Donald L. Trump

**Affiliations:** ^1^ Inova Schar Cancer Institute, Fairfax, Virginia.; ^2^ Inova Medical Center, Falls Church, Virginia.

**Keywords:** carcinoma, pancreatic cancer

## Abstract

**Background:** Pancreatic adenocarcinoma is associated with a very poor prognosis, with a 5 year survival of ∼7.2%. Vitamin D has long been evaluated for benefit as a protective agent and treatment for malignancies. Although cancer incidence and outcomes have been tied to vitamin D levels, there is no clear evidence that supplementation of vitamin D improves outcome in pancreatic cancer to date.

**Case Presentation:** We present a patient who errantly took supratherapeutic doses of vitamin D 50,000 U daily, achieving a serum 25(OH)D level of more than 150 mg/mL, with no appreciable side effects.

**Conclusion:** Her disease was stable for 8 months off of conventional treatment, although it is unclear whether this was related to vitamin D supplementation.

## Background

Pancreatic cancer is the third most common cause of cancer death in the United States, with more than 40,000 deaths per year.^1^ The 5 year survival for all stages of pancreatic adenocarcinoma is ∼7.2%.^2,3^ Reasons for poor outcomes include the advanced state at diagnosis, a location that makes resection difficult, and poor sensitivity to chemotherapy. For this reason, novel approaches are needed to improve outcomes.

Vitamin D has long been evaluated for benefit as a protective agent and treatment for malignancies.^4,5^ Giovannucci et al. demonstrated a statistically significant difference in cancer incidence between those in the bottom decile of predicted 25(OH)D levels and those in the top decile.^6^ Outcomes in colorectal cancer are more favorable for those with higher levels of 25(OH)D.^7^ Although correlation data have been promising, treatment with vitamin D has shown inconsistent results. Increased intake appears to decrease the risk of colon cancer,^8–10^ but has not shown to improve outcomes in other malignancies.^11–13^

Vitamin D impacts cancer cell growth and differentiation.^14,15^ In pancreatic cancer preclinical models, the administration of 1,25(OH)2D analogues causes apoptosis, has antiproliferative effects, and inhibits angiogenesis.^16–18^ The vitamin D receptor (VDR) is involved in multiple metabolic pathways, most notably mineral metabolism, the immune response, and malignancy.^19–22^ The receptor is the mediator of the physiological actions of active forms of vitamin D. Activation of VDR antagonizes the activity of TGFBeta, a promoter of fibrogenesis, correcting maladaptive proinflammatory responses that stimulate oncogenesis.^23–26^

VDRs are ubiquitous within stellate cells in the stroma of pancreatic ductal adenocarcinoma.^27^ Albrechtsson et al. found a more than threefold increase in human cancer cell lines than the normal pancreas.^28^ The stroma, consisting of fibroblastic cells, immune cells, blood vessels, and matricellular proteins, plays a crucial role in promoting and sustaining tumor growth.^29,30^ These tasks are carried out through paracrine stimulation.^31^ In addition, the stroma facilitates resistance to chemotherapy by forming a physical blockade to penetration. In their active state, stellate cells are proinflammatory. However, in a quiescent state, they are producing extracellular matrix at low levels.^32,33^ In murine models, activation of the VDR reduces fibrosis and inflammation. In addition, VDR signaling suppresses paracrine signaling through inflammatory cytokines and growth factors. Sherman et al. treated pancreatic ductal adenocarcinoma (PDA) mice with high doses of calcipotriol, a vitamin D analogue. They found that most mice treated with calcipotriol underwent phenotypic changes that were suggestive of a more quiescent state (i.e., lipid droplet formation).^27^ Sherman et al. demonstrated that VDR is a transcriptional regulator of the stellate cells of the PDA stroma. In addition, the treated mice had a wide array of gene expression, with decreased expression of many genes with a role in metastasis such as CKD1, BIRK5, and CCND1.^27^ The basis for a link between VDR and cancers was bolstered by a genome-wide association study that found that a single SNP in the gene coding for VDR resulted in superior overall survival in pancreatic adenocarcinoma patients.^34^

## Case Presentation

Patient A.N. is an 83-year-old woman who was living in Iran in January of 2015 when she presented to a local hospital with painless jaundice and dark urine. A CT scan showed a pancreatic head mass compressing the common bile duct. For palliation of hyperbilirubinemia, a palliative stent was placed in the common bile duct. A fine needle aspirate obtained through an endoscopic ultrasound (EUS) revealed a poorly differentiated adenocarcinoma. A follow-up CT scan performed on February 13, 2015 revealed a 3.6 × 2.7 cm pancreatic head mass that abutted the superior mesenteric artery and one of its proximal branches. There were suspicious peripancreatic and retroperitoneal nodes and no evidence of distant disease. At the beginning of March, she began taking 50,000 U vitamin D3 (cholecalciferol) daily, ordered directly from the Internet and not under the direction of a healthcare practitioner. Repeat EUS and biopsy on March 3, 2015 again confirmed adenocarcinoma with papillary features. She was referred to an oncologist and surgeon for evaluation. A triple phase CT was done on March 16, 2015 that showed a stable mass without any evidence of involvement of the celiac axis or superior mesenteric artery. Upfront chemotherapy was recommended, with consideration of surgery in the future.

She received gemcitabine/protein nanoparticle-bound paclitaxel in March. On day 10 of cycle 1, she developed neutropenic fever complicated by atrial fibrillation with rapid ventricular response. She was intubated for a brief time who then recovered to her baseline functional status. She was discharged on April 19. Based on her frailty, she was deemed to be a poor candidate for surgery and chemotherapy was not resumed.

She was referred to radiation oncology for consideration of stereotactic body radiotherapy, but she decided to first pursue alternative therapies. She visited an alternative care clinic in April and began taking chelodium, curcumin, community mushroom blend, viscosin, and naltrexone. She also continued to take her daily dose of 50,000 U vitamin D3. Although she was somewhat inconsistent in taking her regimen prescribed by the alternative therapy clinic, she consistently took the same vitamin D3 dose from April until the time of writing.

**FIG. 1.**
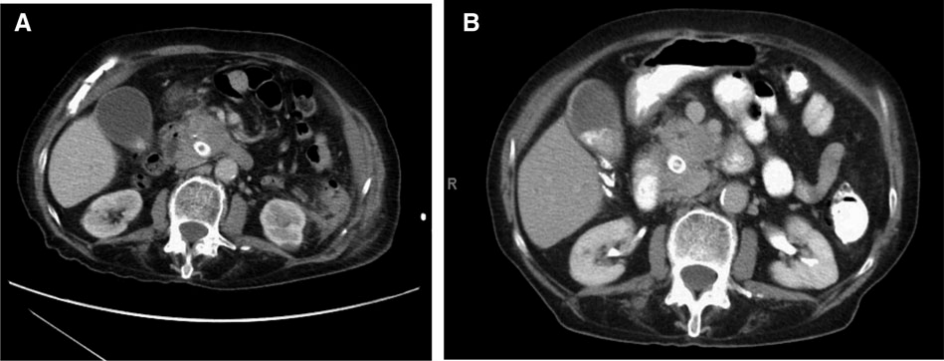
**(A)** February 2015 CT scan. **(B)** October 2015 CT scan.

She was seen first at the Inova Schar Cancer Institute in September 2015. On September 4, 2015, CT scan revealed the pancreatic head mass to be 3.1 × 3.0 cm, slightly smaller than previously with a mild increase in pancreatic duct dilatation. On our first visit on September 5, 2015, we did not obtain a full supplement history, but checked a vitamin D, 25 OH level, which surprisingly was elevated above normal value of >150 ng/mL. Her calcium level was 9.7, which had been 7.7 in March 2015, before starting supplementation. In October, 8 months after her only dose of chemotherapy, she had a CT scan that showed continued disease stability (Fig. 1). As of December 4, 2015, she was stable with no evidence of disease progression by both CT and CA 19-9 level. She was admitted to Fairfax hospital in October of 2015 for diverticulitis with abscess formation that was treated with resection. Currently she describes as feeling quite well with no difficulty accomplishing her activities of daily living.

## Discussion

Several clinical trials are underway that are investigating the use of “high-dose” vitamin D. To our knowledge, no trial is investigating a supplement dose that approaches the level taken by the subject of this case report.^35,36^ For example, NCT01150877, a trial designed to aggressively raise the serum 25(OH)D levels of colorectal cancer patients, aims for a serum (OH)D level of 80–100 ng/mL.^35^ The dose taken by patient A.N. achieved a serum (OH)D level of >150 ng/mL. This dose, initiated through a misunderstanding of the appropriate dose, was very well tolerated with no apparent side effects.

In the phase III MPACT study, the gemcitabine/protein-bound nanoparticle paclitaxel arm had a median progression-free survival (PFS) of 5.5 months.^37^ Patient A.N. had an 8 month PFS despite not even completing one cycle of combination chemotherapy. One cannot conclude that her vitamin D dose was in any way related to this outcome. There is only one CT scan before the initiation of vitamin D, and there is no way to know what her pace of disease would have been in the absence of vitamin D supplementation. In addition, she was taking several other supplements such as shitake mushrooms, although inconsistently and for a shorter duration, which were also intended to treat her malignancy.

Nonetheless, given the poor prognosis of pancreatic cancer and the limited treatment options for patients, this case should stimulate further investigation. The daily dose of 50,000 U of vitamin D3 was well tolerated in our patient for over 10 months at the time of writing. Consideration should be given to a clinical trial that evaluates a similar dose. Correlatives may include an evaluation of gene expression changes as well as phenotypical changes in the stroma.

## Learning Points

• Although epidemiological data have been suggestive of a correlation between vitamin D levels and cancer incidence, there is no evidence of a positive impact of vitamin D supplementation on pancreatic cancer outcome.
• Vitamin D impacts the stroma in murine models of pancreatic cancer, resulting in a more quiescent phenotype.
• The patient in the case report tolerated a dose of 50,000 U of vitamin D3 over an ∼10 month period.
• Vitamin D at high doses should be investigated as a novel therapeutic agent for pancreatic adenocarcinoma.

## Abbreviations Used

PDA: pancreatic ductal adenocarcinoma
PFS: progression-free survival
SNP: single nucleotide polymorphism
VDR: vitamin D receptor
